# To Contributors

**Published:** 1891-02

**Authors:** 


### TO CONTRIBUTORS.

It has been decided to change the date of issue of this journal from the 15th of each month to the 1st. It will, therefore, be necessary to have all contributions placed in the hands of the editor not later than the 10th of the month.

